# Blue-Violet Laser Modification of Titania Treated Titanium: Antibacterial and Osteo-Inductive Effects

**DOI:** 10.1371/journal.pone.0084327

**Published:** 2013-12-17

**Authors:** Takanori Kawano, Widyasri Prananingrum, Yuichi Ishida, Takaharu Goto, Yoshihito Naito, Megumi Watanabe, Yoritoki Tomotake, Tetsuo Ichikawa

**Affiliations:** Department of Oral & Maxillofacial Prosthodontics and Oral Implantology, The University of Tokushima, Institute of Health, Biosciences, Tokushima, Japan; University of Rochester, United States of America

## Abstract

**Background:**

Many studies on surface modifications of titanium have been performed in an attempt to accelerate osseointegration. Recently, anatase titanium dioxide has been found to act as a photocatalyst that expresses antibiotic properties and exhibits hydrophilicity after ultraviolet exposure. A blue-violet semiconductor laser (BV-LD) has been developed as near-ultraviolet light. The purpose of this study was to investigate the effects of exposure to this BV-LD on surface modifications of titanium with the goal of enhancing osteoconductive and antibacterial properties.

**Methods:**

The surfaces of pure commercial titanium were polished with #800 waterproof polishing papers and were treated with anatase titania solution. Specimens were exposed using BV-LD (λ = 405 nm) or an ultraviolet light-emitting diode (UV-LED, λ = 365 nm) at 6 mW/cm^2^ for 3 h. The surface modification was evaluated physically and biologically using the following parameters or tests: surface roughness, surface temperature during exposure, X-ray diffraction (XRD) analysis, contact angle, methylene blue degradation tests, adherence of *Porphyromonas gingivalis*, osteoblast and fibroblast proliferation, and histological examination after implantation in rats.

**Results:**

No significant changes were found in the surface roughness or XRD profiles after exposure. BV-LD exposure did not raise the surface temperature of titanium. The contact angle was significantly decreased, and methylene blue was significantly degraded. The number of attached *P. gingivalis* organisms was significantly reduced after BV-LD exposure compared to that in the no exposure group. New bone was observed around exposed specimens in the histological evaluation, and both the bone-to-specimen contact ratio and the new bone area increased significantly in exposed groups.

**Conclusions:**

This study suggested that exposure of titanium to BV-LD can enhance the osteoconductivity of the titanium surface and induce antibacterial properties, similar to the properties observed following exposure to UV-LED.

## Introduction

Photocatalysis, as exemplified by titanium dioxide (TiO_2_), was originally developed by Fujishima and Honda[1], and the effect has been applied to various fields ever since. Wang et al. reported the photogeneration of a highly amphiphilic TiO_2_ surface, i.e., with both hydrophilic and oleophilic effects[2]. Recently, the ultraviolet (UV) light-induced photocatalytic activity of titanium has attracted considerable attention in environmental and clean-energy sciences[3-6]. Titanium is highly biocompatible, and both pure titanium and titanium alloys have been used in the medical field, particularly in orthopedics and as dental implants. Aita et al. reported that pretreatment of titanium with UV light substantially enhances its osteoconductive capacity in association with UV-catalytic progressive removal of hydrocarbons from the TiO_2_ surface, resulting in photofunctionalization of titanium and thereby enabling more rapid and complete establishment of bone-titanium integration[7]. On the other hand, exposure of titanium surfaces to UV also result in changes to the microbial growth properties of the metal[8,9]. Indeed, photocatalysis as exemplified by TiO_2_ has 2 major effects: oxidation and hydrophilicity[10]. It is generally accepted that hydrophilicity of the titanium surface will enhance rapid bone contact, while strong oxidation due to photocatalysis will result in cytotoxic activity and bactericidal effects[11,12]. However, the biological responses of these two opposite effects, i.e., oxidation and hydrophilicity on cells and bacteria, have not been investigated under the same conditions.

In the clinical setting, UV light-aided treatment of titanium has been mainly studied in the context of manufacturing medical devices that will be implanted within the human body[7,13-15]. Therefore, reactivation of the titanium implant at the bedside/chairside or in the oral cavity is also necessary. Exposure of a limited area and development of a mobile apparatus are needed to achieve this goal. A low-pressure mercury lamp that emits germicidal UVC light (shorter than 280 nm) has been conventionally used as a light source for this purpose. Moreover, UV light-emitting diodes (UV-LEDs) with a wavelength of 365 nm have been developed and offer the following advantages: low cost, energy efficient, long life, easy control of emission, and no production of mercury waste[15,16]. Visible blue-violet lasers with a wavelength of 405nm have also developed, and such devices may facilitate bedside/chairside use of light sources for modification of titanium.

The purpose of this study was to examine the effects of a visible blue-violet laser and anatase titania solution on titanium, particularly the bone and microbiological response, and to determine the potential of titanium treatment at the bedside. 

## Materials and Methods

### Titanium specimens and light sources and exposures

Commercial pure titanium (grade II) was used for the specimens. Three types of specimens, i.e., disk plate (φ33 mm × 1 mm) for the contact angle test and methylene blue degradation test, square plates (10 mm × 10 mm × 2 mm) for the cell proliferation test and bacterial adherence test, and cylindrical rods (φ2 mm × 5 mm) for the animal test, were prepared. The specimens were serially ground and manually polished with #800 abrasive silica papers and ultrasonically cleaned with acetone, ethanol, and distilled water for 10 min progressively. Specimens were then dried in a desiccator for 12 h without light. In addition, 30 μL of anatase titania solution (TITANNEXT 21, 0.85%, Marutomi, Gifu, Japan) was used to evenly coated the irrigated titanium surface according to methods described in a standardized manual, and samples were then dried in the desiccator for 12 h without light. Five specimens were prepared for every condition.

Two light sources, blue-violet semiconductor laser (BV-LD; λ = 405 nm; 1.0 W; NUV102E, NICHIA, Anan, Japan) and UV-LED (λ = 365 nm; 0.31 W; NCSU033A(T), NICHIA, Anan, Japan) were employed for modification of the titanium surface. The surfaces of specimens were exposed to each light at an intensity of 6.0 mW/cm^2^ for 3 h.

### Physical evaluation

#### Roughness of the modified surfaces

Average surface roughness (Ra) was measured using a surface measuring device (HANDYSURF, Tokyo Seimitu, Tokyo, Japan). Three areas for each disk specimen and one lateral side of each cylindrical rod specimen were measured; the data were averaged.

#### Structure of the modified surfaces

The surface crystalline structure of the specimens was analyzed using X-ray diffraction analysis (XRD; MINIFLEX, RIGAKU, Tokyo, Japan).

#### Surface temperature during exposure

A thermocouple (C520-13K, CHINO, Tokyo, Japan) was fixed tightly in the tube (φ2.0 mm × 4.0 mm), which was made of a copper plate (0.3 mm in thickness) and was fixed on the center of the specimens by soldering. The temperature of the center of the specimen during exposure was measured with a thermocouple and temperature monitoring device (NR-250, Keyence, Osaka, Japan).

#### Hydrophilicity

Static contact angles were measured using a contact angle measuring device (IMC-159D, IMOTO, Tokyo, Japan) before/after each light exposure.

#### Photocatalytic effects

Photocatalytic effects were evaluated by the degradation of methylene blue. In this assay, 10 μL of 0.2% methylene blue solution (MB) was dropped onto 5 points of the specimen. After exposure to each light source, the specimens were soaked in 10 mL distilled water to allow the MB on the specimen to dissolve. After pipetting 30 times, the absorbance of the solution was measured using a spectrophotometer (Novaspec II, Amersham Pharmacia Biotech, Cambridge, UK) at 664 nm. 

### Biological evaluation

#### Antibacterial properties

*Porphyromonas gingivalis* (ATCC 33277), a pathogenic bacterium involved in peri-implantitis, was employed. The bacterial strain was cultured for 72 h, and colonies were resuspended in phosphate-buffered saline (PBS) to 1.0 × 10^7^ cfu/mL as the final concentration. 

Sterilized saliva was prepared by sterilization using membrane filters, and the specimens were then soaked in the saliva for 10 min to allow the pellicle to be formed on the specimens. After rinsing in PBS 3 times, 30 μL of the bacterial suspension was dropped on each specimen, and specimens were then maintained at room temperature for 2 h. After rinsing again in PBS to remove loosely adhered microorganisms, the specimens were fixed in 2 mL glutaraldehyde solution, formaldehyde solution, and PBS for 1 h at room temperature and then rinsed with PBS 3 times and dehydrated with a series of ethanol solutions. All samples were dried to the critical point, coated with gold using an ion coater (IB-3, Eiko Engineering, Hitachinaka, Japan), and observed by scanning electron microscopy (Miniscope TM-1000, Hitachi, Tokyo, Japan). Five representative areas of each specimen were photographed at a magnification of 5,000×. Finally, the bacteria attachment ratio was calculated by dividing the number of adhered cells in the exposed specimens by that in the nonexposed specimens.

#### Cell proliferation

MC3T3-E1 cells (osteoblastic cells, Riken, Ibaragi, Japan ) were cultured in α-MEM (Wako, Osaka, Japan) supplemented with 10% fetal bovine serum (FBS; Japan Bioserum, Tokyo, Japan), 100 U/mL penicillin, and 100 μg/mL streptomycin (Sigma Aldrich Japan, Tokyo, Japan). NIH3T3 cells (fibroblastic cells, Riken) were cultured in DMEM (1000 mg/L glucose; Wako) supplemented with 10% FBS, 100 U/mL penicillin, and 100 μg/mL streptomycin. All *in vitro* cell incubations were performed in a CO_2_ incubator (Binder, CB 150/APT-line) at 37°C in 5% CO_2_ atmosphere with 100% relative humidity.

Titanium specimens were placed in 24-well polystyrene plates. When cells reached 70% confluence, they were detached by trypsinization and seeded onto the specimens at a density of 1.3 × 10^5^ cells/mL. After 48 or 96 h, the specimens were transferred to new plates containing fresh medium. Cell proliferation assay reagent (Cell Counting Kit-8, Dojindo Laboratories, Kumamoto, Japan) was added at a concentration of 100 μL/mL. After incubation at 37°C for 3 h in a humidified CO_2_ incubator, absorbance at 450 nm was measured using a microplate reader (Multiskan JX Version 1.1, Thermo Labsystems, Thermo Fisher Scientific, Kanagawa, Japan). 

#### Animal experiments

Ten 12-week-old male Wistar rats (Charles River Laboratories Japan, Yokohama, Japan) were used in this study. The rats were divided into 2 groups (BV-LD and UV-LED exposure, 5 rats in each group). The rats were anesthetized with 1%–2% pentobarbital sodium (Somnopentyl, Kyoritsu, Tokyo, Japan), and the implant site was prepared 10 mm from the distal edge of the left femur by drilling with a φ2 mm pilot bur after determining the site using a round bur. Then, control specimens were placed into the left legs of rats, while irradiated specimens were placed into the right legs. Every specimen was inserted into the implantation site such that the gap between the upper end of the specimen and the femur bone surface disappeared. Four weeks after implantation, rats were sacrificed using an overdose of pentobarbital sodium. Specimens were excised with surrounding tissue and preserved in 10% formalin neutral buffer solution for 1 week. After polymerization, the embedded specimens were cut into 200-μm-thick sections using a microcutter (MC201, Maruto, Tokyo) parallel to the axis of the femur bone. Slices were then stained with 0.1% toluidine blue solution for histological observation. Typical specimens were chosen from those that contained the center region of the cylindrical specimens. Their histological states were recorded for measurement of the bone-to-specimen contact ratio (%), and new bone area around the specimens was measured by the method adopted before [17] using NIH Image 1.55 software (National Institute of Health, USA). 

The Animal Care and Use Committee of the University of Tokushima approved this protocol, and all experiments were performed in accordance with the Guidelines for the Care and Use of Lab Animals at the University of Tokushima.

### Statistical analysis

Significant differences were determined using analysis of variance (ANOVA) followed by Tukey post-hoc tests for multiple comparisons. 

## Results

### Physical evaluation

**Figure 1:**
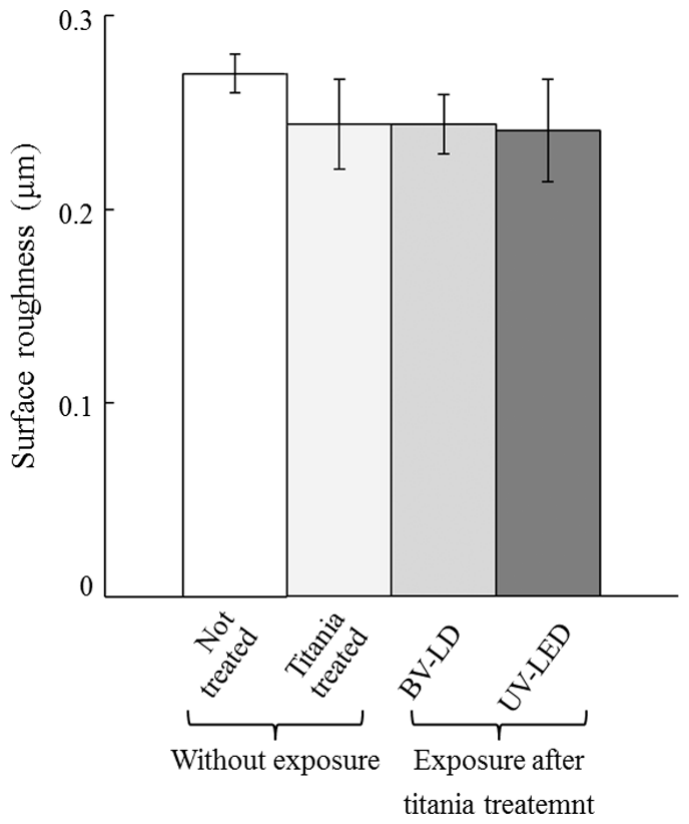
Changes in the average surface roughness of each specimen.

**Figure 2:**
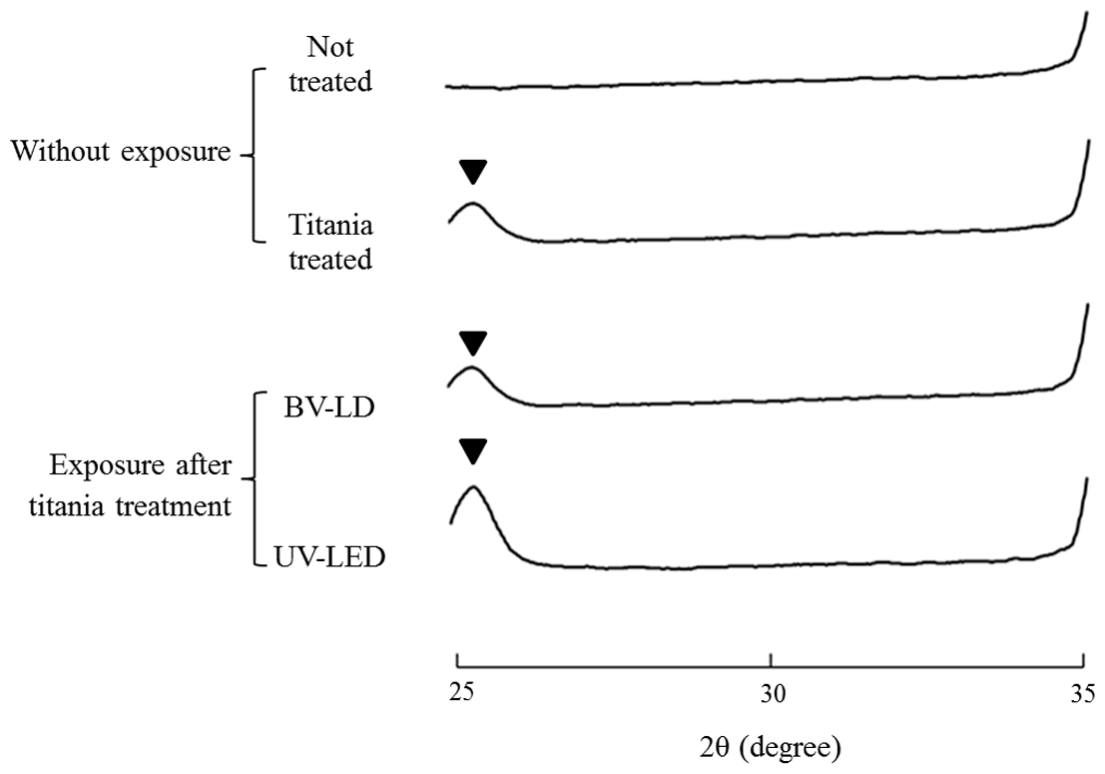
Changes in the XRD patterns of each specimen.

**Figure 3:**
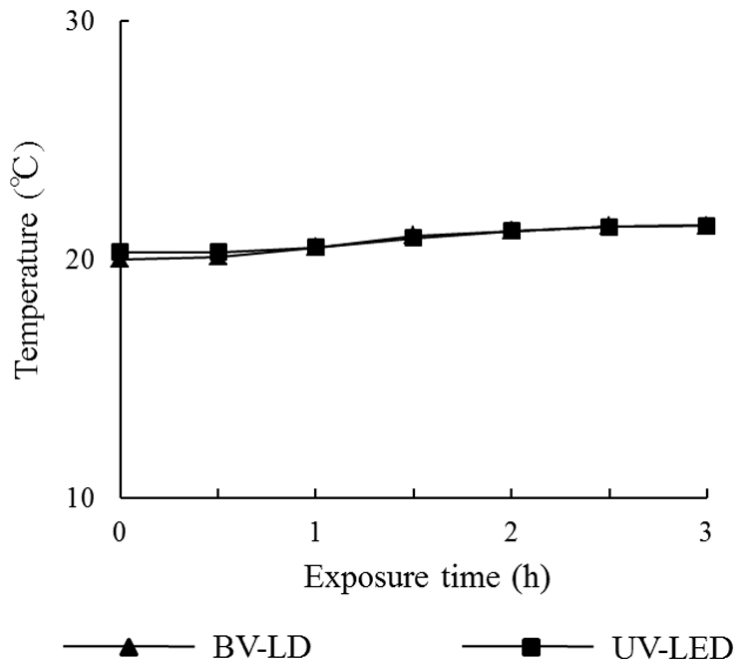
Temperature changes on the surface during each exposure.

**Figure 4:**
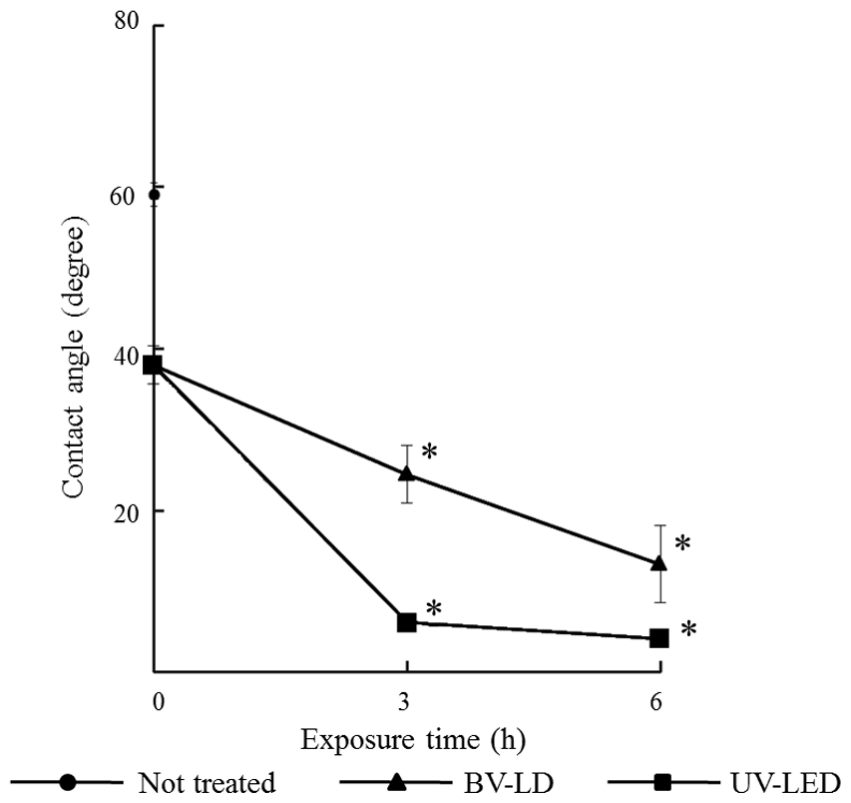
Changes in the contact angle of each specimen after titania treatment.

**Figure 5:**
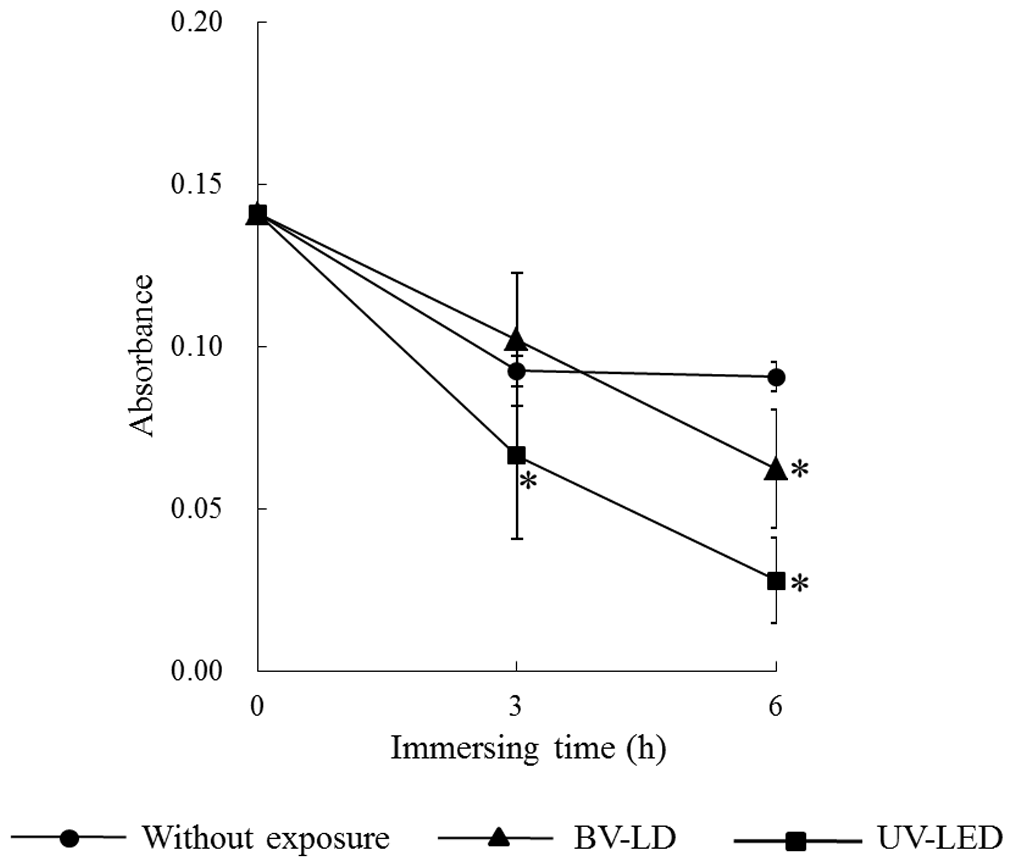
Changes in the absorbance of methylene blue staining for each specimen after titania treatment.

First, we evaluated the characteristics of titanium following exposure to light. The average surface roughness (Ra) of anatase titania solution-treated titanium was significantly lower than that in untreated titanium (Figure 1). However, no significant differences were found between specimens exposed to light and those not exposed to light. XRD patterns revealed typical pure anatase peaks in anatase titania solution-treated titanium (Figure 2). Exposure of the specimen to each light source enhanced this anatase peak. Moreover, the surface temperature of specimens varied only slightly during 3-h BV-LD and UV-LED exposure, but never increased over 36°C (Figure 3). We also measured static contact angles before and after exposure to light (Figure 4). Before exposure, contact angles on non–titania-treated and titania-treated specimens were 59° and 38°, respectively. UV-LED decreased the contact angle down to 3° with 3 h, and BV-LD exposure also gradually decreased the contact angle with increasing exposure time. Finally, in the test of methylene blue degradation, absorption decreased monotonically after UV-LED and BV-LD exposure (Figure 5). 

### Biological evaluation

**Figure 6:**
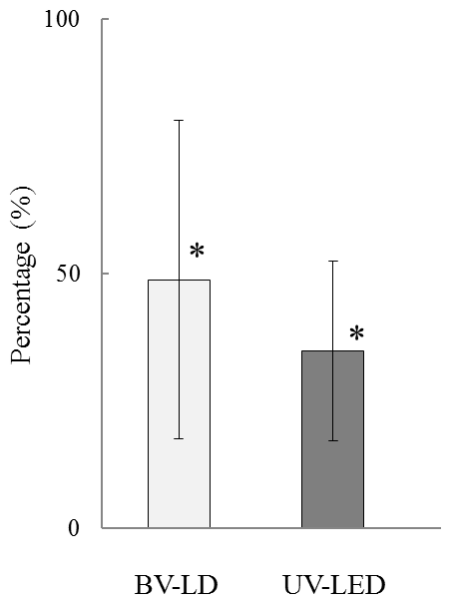
Adherence of *Porphyromonas gingivalis* to each specimen. Percent adherence with exposure to that without exposure after titania treatment.

**Figure 7:**
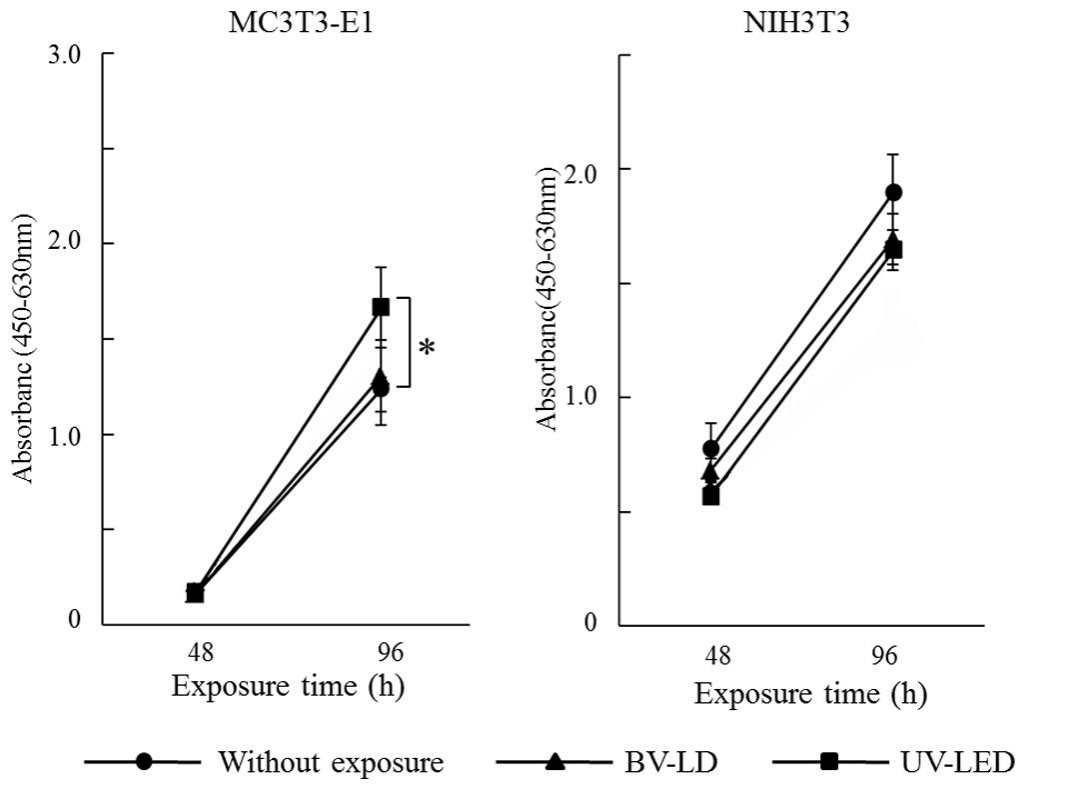
Chronological changes of cell proliferation in each specimen after titania treatment.

Next, we investigated the biological properties of light-treated titanium. As shown in Figure 6, the P. *gingivalis* adherence ratio decreased by 49% and 35% following BV-LD and UV-LED exposure, respectively. Additionally, both osteoblasts and fibroblasts grew on all specimens with time (Figure 7). UV-LED exposure significantly enhanced the proliferation of MC3TC-E1 cells at 96 h, whereas BV-LD had less of an effect on cell proliferation. All light exposures also had minimal effects on cell proliferation in NIH3T3 cells. 

**Figure 8:**
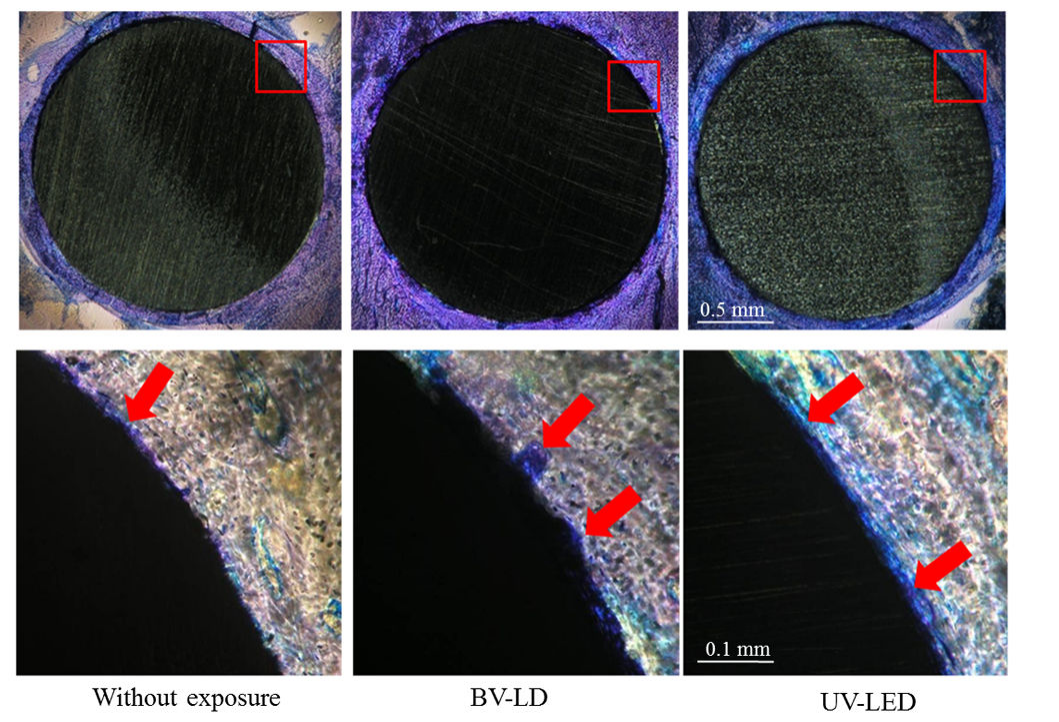
Histological views 1 month after implantation of titania-treated specimens. Arrows: new bone formation.

**Figure 9:**
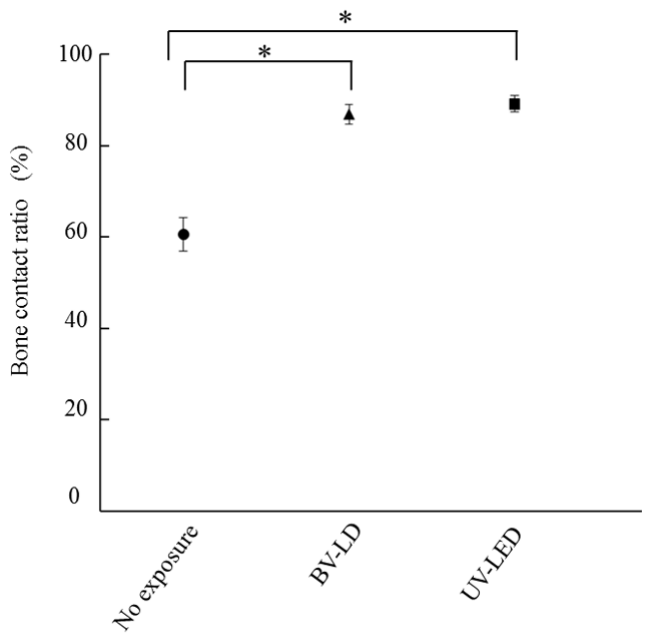
Bone contact ratio 1 month after implantation of titania-treated specimens.

**Figure 10:**
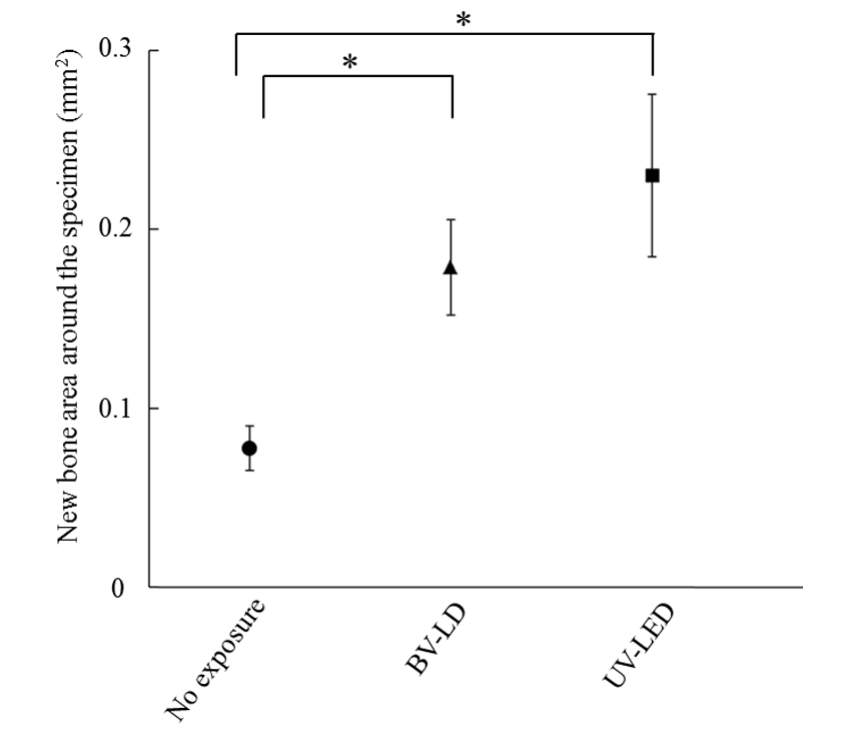
New bone formation area around the specimen 1 month after implantation of titania-treated specimens.

We then analyzed the osteoconductive properties of the treated titanium specimens. Interestingly, histological analysis of bone around each specimen at 1 month after specimen placements revealed an increase in new bone around specimens that had been exposed to BV-LD or UV-LED (Figure 8). Additionally, the bone contact rate of the specimen without exposure was 60%, and BV-LD and UV-LED exposure increased the bone contact rate to 86% and 89%, respectively (Figure 9). Compared to unexposed specimens, these differences were statistically significant; however, there was no significant difference between the light sources. Finally, we found that light exposure increased new bone formation around the titanium specimen (Figure 10). The area of new bone formation before the exposure was less than 0.1 mm^2^, but increased to more than 0.2 mm^2^ after BV-LD or UV-LED exposure. Again, these differences were significant compared to the unexposed control, but no significant differences were observed between light sources. 

## Discussion

Titanium dioxide, particularly in the anatase form, is a photocatalyst under UV light. Many reports have investigated the biological response to titanium following UV exposure, and these studies have shown that bone-titanium integration can be more rapidly and completely established following UV treatment [7,9,13-15]. Aita et al. stated that the osseo-inductive capacity of the titanium surface depends on the UV-dose responsive removal of hydrocarbons from the TiO_2_ surface, but not on the hydrophilic status [7]. In addition, it has been suggested that UV-C, with a wavelength of 250 nm, is more effective than UV-A, with a wavelength of 360 nm. Thus, modification of the titanium surface depends on the energy of the UV exposure and the chemistry of the TiO_2_. 

In contrast, semiconductor light sources, i.e., lasers and LEDs, are convenient to use in clinical settings because they are compact, allow for directionality, and have a long light-source lifetime [16,18]. In addition, UV-A and visible light are more preferable due to biological hazards; UV-C damages cellular DNA, while UV-A attacks the cell though generation of radicals [19,20] .

In this study, we attempted to modify the titanium surface (grade II) to improve biocompatibility for clinical use. The application of a specific anatase titania solution and LD/LED exposure were combined, and the surface was photocatalytically activated. Medical devices usually use grade IV titanium and titanium alloy, not grade II titanium that was used in this study. The grade of titanium used affects less characteristics of the thin anatase layer formed on the titanium surface. Generally, the wavelength is required to be less than 400 nm to act as a photocatalyst on anatase-type TiO_2_. Photocatalysis is also increased by exposure to visible light, with varying efficiencies. In this study, we examined whether near-ultraviolet exposure, i.e., BV-LD, which has a wavelength of 405 nm, had both antimicrobiological and osteoconductive effects, similar to the effects of UV exposure on the titanium surface. 

The application of anatase-titania solution decreased the contact angle and enhanced the crystal structure of anatase on the titanium surface. Interestingly, no changes in the physical nature of the titanium surface (roughness, temperature, and structure) were observed after exposure to BV-LD or UV-LED. The contact angle lineally decreased with the increasing exposure time and eventually became less than 10°, indicating a change to a superhydrophilic state. Photocatalytic effects, as demonstrated by methylene blue degradation, were also increased with prolonged exposure time. In these tests, we observed that the effects of BV-LD exposure were a slightly inferior those of UV-LED exposure.

We also examined the influence of light-exposed titanium surfaces on hydrophilic and photocatalytic effects both in vitro and in vivo. First, the antibactericidal effects of the modified titanium surface were evaluated using *P. gingivalis* in a bacterial adhesion assay. *P. gingivalis* is an important bacterium causing in peri-implant and periodontal diseases. BV-LD and UV-LED exposures decreased bacterial adherence on the titanium surface. Photocatalysis depends on the ability of the catalyst to create electron-hole pairs, which generate free radicals able to undergo secondary reactions. 

The proliferation of osteoblasts on the exposed specimens was significantly increased compared to that on unexposed specimens, whereas no change was observed in the proliferation of fibroblasts, regardless of exposure. Changes in the hydrophilicity of the specimen could enhance osteoblast adherence, while the adherence of *P. gingivalis* appeared to be unassociated with the hydrophilicity of the material. Hence, this difference between bacteria and mammalian cells may be related to the potential protective mechanisms the different cell types have to combat the formation of toxic radicals [21-23].

Finally, histological and quantitative analyses of bone formation suggested that UV-LED/BV-LD exposure enhanced new bone formation compared to unexposed specimens. These results are in agreement with many reports on the effects of UV exposure to the titanium surface [7,13]. 

In conclusion, our data suggested that exposure to BV-LD can enhance the osteoconductivity of a titanium surface and induce antibacterial　properties similar to the effects observed after exposure to UV-LED. However, the exposure time used in this study is too long for clinical use due to the low exposure strength (6 mW/cm^2^). Thus, for clinical use, the exposure time could be shortened by increasing the current to the laser and LED and focusing the exposure area. 
